# The Long-Term Maintenance of Upper Limb Motor Improvements Following Transcranial Direct Current Stimulation Combined with Rehabilitation in People with Stroke: A Systematic Review of Randomized Sham-Controlled Trials

**DOI:** 10.3390/s21155216

**Published:** 2021-07-31

**Authors:** Víctor Navarro-López, Manuel del Valle-Gratacós, Rubén Fernández-Matías, María Carratalá-Tejada, Alicia Cuesta-Gómez, Francisco Molina-Rueda

**Affiliations:** 1 Motion Analysis, Biomechanics, Ergonomy and Motor Control Laboratory (LAMBECOM Group), Physical Therapy, Occupational Therapy, Rehabilitation and Physical Medicine Department, Health Sciences Faculty, Rey Juan Carlos University, 28922 Alcorcón, Spain; victor.navarro@urjc.es (V.N.-L.); alicia.cuesta@urjc.es (A.C.-G.); francisco.molina@urjc.es (F.M.-R.); 2 Téxum S.L. Physiotherapy Center, 28821 Coslada, Spain; manuel.valleg@edu.uah.es; 3 Research Unit, Hospital Universitario Fundación Alcorcon, 28922 Madrid, Spain; ruben.fernandezm@edu.uah.es; 4 Research Institute of Physiotherapy and Pain, University of Alcala, 28801 Madrid, Spain

**Keywords:** physical therapy, rehabilitation, stroke, transcranial direct current stimulation, upper limb

## Abstract

Background: The effectiveness of transcranial direct current stimulation (tDCS) in the upper limb (UL) motor rehabilitation of stroke has been widely studied. However, the long-term maintenance of its improvements has not yet been proven. Methods: A systematic search was conducted in MEDLINE/Pubmed, Web of Science, PEDRo, and Scopus databases from inception to April 2021. Randomized controlled trials were included if they performed a tDCS intervention combined with UL rehabilitation in stroke patients, performed several sessions (five or more), and assessed long-term results (at least three-month follow-up). Risk of bias and methodological quality were evaluated with the Cochrane RoB-2 and the Oxford quality scoring system. Results: Nine studies were included, showing a high methodological quality. Findings regarding UL were categorized into (1) functionality, (2) strength, (3) spasticity. All the studies that showed significant improvements retained them in the long term. Baseline functionality may be a limiting factor in achieving motor improvements, but not in sustaining them over the long term. Conclusion: It seems that the improvements achieved during the application of tDCS combined with UL motor rehabilitation in stroke were preserved until the follow-up time (from 3 months to 1 year). Further studies are needed to clarify the long-term effects of tDCS.

## 1. Introduction

Cerebrovascular accident is defined by the World Health Organization as “the set of rapidly progressive clinical signs due to a focal, sometimes global, alteration of brain function that lasts more than 24 h or causes death without any other apparent cause than its vascular origin” [1]. The abnormal neurorepair factor that occurs after a stroke, as well as the limitations of functional recovery that arise after rehabilitation protocols, cause alternatives to be considered in order to increase the margin for improvement of the patient by increasing the modulation on cortical plasticity [2]. Noninvasive brain stimulation techniques (NIBS) have potential utility to control and modulate the excitability of intracortical neuronal circuits [2], maintaining their effect compared to stimulation time [3], which is one of the outstanding justifications for their use. The application of transcranial direct current stimulation (tDCS) seems to modify the discharge threshold of cortical neurons, without the need to apply high intensities, since with low-amplitude currents (0.5–2 mA), it penetrates cortical tissues [4]. tDCS has the function to modulate cortical excitability in a polarity-dependent manner, since cathodic stimulation decreases cortical excitability, while anode stimulation increases it. Even so, it is necessary to consider the great interindividual variability [4], as well as the dependence of the activity levels of the stimulated tissues [5,6]. The tDCS represents a relatively inexpensive, simple, and portable technique, with great potential for use in the rehabilitation of stroke [7,8].

Studies carried out with tDCS suggest that, in any of its assemblies, this intervention may improve motor function and the functionality in activities of daily life after a stroke [9], being a potentially useful and safe rehabilitation tool for the upper limb (UL) motor recovery in people following stroke [10,11]. The tDCS produces mechanisms like long-term potentiation (LTP). It has been hypothesized that these changes can be due to modifications at the dendrite level (glutamatergic receptors such as the n-methyl-D-aspartic receptor, NMDA) [12] and an enhancement of brain-derived neurotrophic factor (BDNF) [13] release. However, these modifications induced by tDCS in isolation do not lead to significant and permanent synaptic changes if they are not combined with voluntary activity such as rehabilitation [14]. Although the physiological changes involved in LTP are being investigated, it is unclear whether the motor and functional gains of UL following tDCS in combination with rehabilitation in poststroke individuals are sustained over the long term (3 months or longer). For this reason, a systematic review is needed to clarify its efficacy [15,16]. Therefore, this review aims to determine whether the improvements achieved in upper limb function during tDCS and rehabilitation are diluted or maintained after cessation of tDCS.

## 2. Materials and Methods

### 2.1. Design

This review was reported following the PRISMA recommendations for reporting systematic reviews [17] (Table S1 in Supplementary Material). The review protocol was not registered due to delays with registrations in PROSPERO caused by the SARS-CoV-2 pandemic.

### 2.2. Search Strategy and Database

To carry out the bibliographic search, the following databases were consulted on 15 April 2021: Medline/PubMed, PEDRo, Scopus, and Web of Science. The following keywords were used in combination with Boolean operators: “tDCS”, “transcranial-direct-current-stimulation”, “stroke”, “cerebrovascular accident”, “upper-limb”, “hand”, “upper extremity”, “rehabilitation”, and “physical therapy” (Table S2 in Supplementary Material).

### 2.3. Screening Process and Eligibility Criteria

The title and abstract were evaluated by two different researchers (VNL; MdVG) and discrepancies were resolved by a third researcher (FMR). The same process was conducted for full-text screening.

Inclusion criteria: The literature search was limited to randomized clinical trials (RCT) and pilot randomized controlled trials in English and Spanish that carried out a tDCS intervention in combination with rehabilitation (including physical therapy (PT) or occupational therapy (OT)) of the UL in stroke patients, performed several sessions (five or more sessions), and measured the long-term results (at least three months of follow-up). Studies were not limited by year of publication.

Exclusion criteria: studies were excluded if they did not analyze measures of the UL, if they did not include at least one control group treated with tDCS and a placebo group, and if they included pathologies other than stroke.

### 2.4. Data Extraction

Standardized methodology was used to extract data from studies that met the inclusion criteria. Data on the first author, year of publication, design, number of patients, type of measurement tools, type of therapy applied, protocol for tDCS application, electrode placement, and study results were extracted.

### 2.5. Methodological Quality Assessment and Risk of Bias

In order to analyze the methodological quality of each individual study, the Oxford quality scoring system was used [18]. This scale includes items related to randomization, masking, and the description of the losses to follow-up, with the highest score (highest methodological quality) being 5 and the lowest score (lowest methodological quality) being 0 [18]. In order to analyze the risk of bias of each individual study, the RoB-2 (the revised Cochrane risk-of-bias tool for randomized trials) was used [19]. It is a valid tool that evaluates domains related to random sequence generation, allocation concealment, blinding of participants and personnel, blinding of outcome assessment, incomplete outcome data, selective reporting, and other bias. Each item may be classified as a risk of bias that is “high risk”, “low risk”, or “some concerns” [19]. The entire methodological process was carried out by two different researchers, and any discrepancy was resolved by a third researcher.

## 3. Results

**Figure 1:**
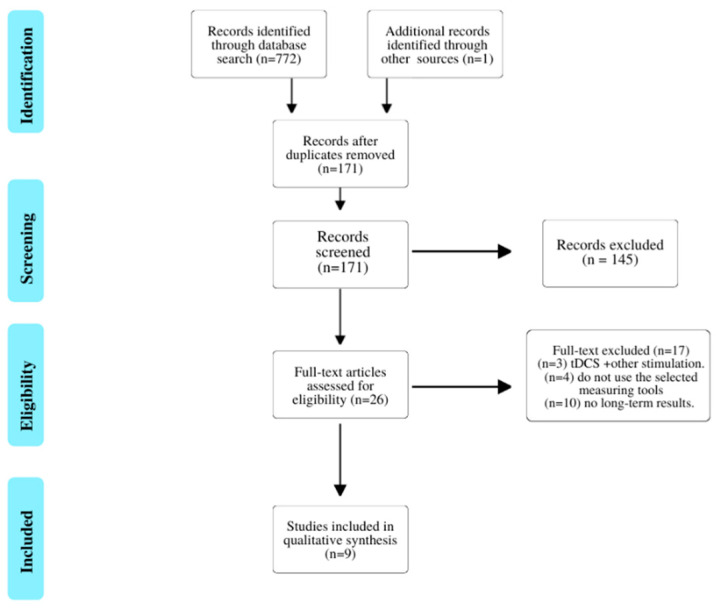
Flowchart of the selection process.

A total of 773 studies were retrieved. Duplicate studies were eliminated, leaving a total of 171 studies, on which a critical reading of the title and abstract was carried out. After the first screening, there was a total of 26 studies, which were obtained and read in full text together with three studies included through reading the bibliography of two systematic reviews. Finally, 9 studies [20,21,22,23,24,25,26,27,28] were included in the review after performing a second screening, with a total of 368 subjects (255 men/113 women). The whole screening process is shown in the PRISMA flow diagram (Figure 1).

### 3.1. Quality Assessment

**Figure 2:**
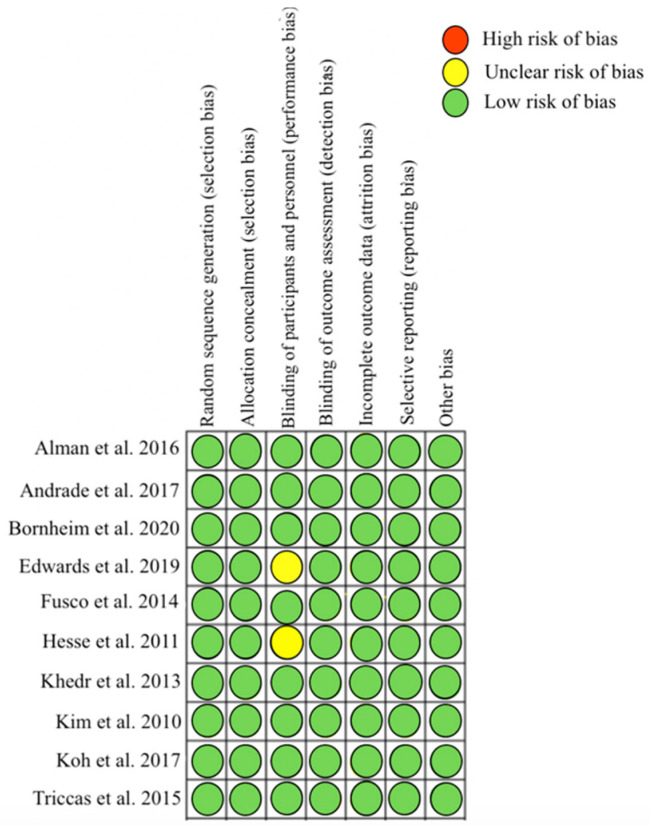
Risk of bias of included studies.

Regarding methodological quality, of the nine included studies, eight [20,21,22,23,25,26,27,28] showed “high” (5 points) methodological quality according to the Oxford quality scoring system, and one study [24] showed a methodological quality rated as “moderate” (score of 4) (Table S3 in Supplementary Material). All studies were double-blind. Regarding the risk of bias, seven studies showed low risk in all measured domains [20,21,23,25,26,27,28] (selection bias, performance bias, detection bias, attrition bias, reporting bias, and other bias). The other two studies [22,24] showed some concerns regarding blinding of participants and personnel (Figure 2).

### 3.2. Study Characteristics

The individual characteristics of each study are summarized in Table 1.

#### 3.2.1. Subject and Studies

The years of completion of the studies span from 2011 to 2020. Regarding the follow-up time of the results, five studies followed up for three months [20,23,24,25,28], three followed up for six months [22,26,27], and one followed up for one year [21]. The design of the studies was RCT, all being double blind except for one triple blind [21]. Among the studies, one performed a parallel RCT [21] and another was a pilot RCT [25].

Of the 368 subjects (255 men/113 women), therapy was performed in stroke patients of all stages; two studies included acute patients [21,24], four included subacute patients [23,24,25,26,28], and four included chronic patients [20,22,27,28]; mean time since injury was 25.93 months. The mean age of the subjects was 60.86 years. Studies included people with ischemic and hemorrhagic strokes of the cortical (left or right) and subcortical territory.

Subjects’ functionality was assessed at baseline in most studies using the Fugl-Meyer Upper Extremity Test (UEFM) [20,22,23,24,26,27,28], presenting a range of functionalities between 4–70 points; the mean score on the UEFM was higher than 30 in two studies [20,26], and lower than 30 in five studies [23,24,25,27,28]. One study used the Wolf Motor Function Test (WMFT) [21] and two studies used the hand-grip force [21,25] (Table 1).

#### 3.2.2. Treatment

The applied treatment methods among the included studies were varied: five applied tDCS and PT [20,21,23,25,27], among which two studies also performed OT intervention [21,27]; three studies performed PT intervention based on robotic arm training [22,24,28]; and one applied OT. Regarding the method of application of tDCS, two variables were differentiated: one was the type of stimulation applied (anodic, cathodic, or bi-hemispheric) and the other was the time of application of rehabilitation (during or after tDCS). Regarding the type of stimulation, three performed anodic tDCS [20,21,28], one performed bi-hemispheric tDCS [27], one performed cathodic tDCS [23], and three made a comparison between the different application variables [24,25,26].

According to the time of application of rehabilitation, seven [20,21,22,23,25,27,28] studies applied the tDCS before the therapy, one study applied it simultaneously [24], and one study applied it before and after rehabilitation [26]. Regarding the intensity, time of application of the tDCS, and number of sessions, three studies applied an intensity of 1 mA [20,21,28], two studies applied an intensity of 1.5 mA [23,27], and four studies applied an intensity of 2 mA [22,24,25,26]. The current density applied varied depending on the size of the electrodes and the current intensity (current density: mA/cm^2^). Six studies used 35-cm^2^ electrodes [20,22,23,24,25,28], while 3 studies used 25-cm^2^ electrodes [21,26,27]. The current density ranged between 0.028–0.08 mA/cm^2^, being 0.028 mA/cm^2^ in 2 studies [20,28], 0.04 in one study [21], 0.043 in one study [23], 0.057 in three studies [22,24,25], 0.06 in one study [27], and 0.08 in one study [26].

One study applied a 10-min stimulation [23], six performed a 20-min stimulation [20,21,22,24,26,28], one performed a 25-min stimulation [25], and one performed a 30-min stimulation [27]. The number of sessions varied from 6 to 30, with each study applying a different number of sessions (Table 1).

#### 3.2.3. Measurement Tools

Primary and secondary measures applied in the included studies related to the UL were reflected. Among the nine included studies, eight analyzed the functionality of the UL [20,21,22,23,24,26,27,28], four analyzed the muscular strength of the UL [22,23,24,25], and three analyzed the spasticity of the UL [21,24,27]. The measurement tools used are detailed in Table 1.

### 3.3. Study Results

The results of the studies that obtained significant improvements both at the end of the study and at follow-up will be shown below. All the studies that showed significant improvements in favor of the use of tDCS in combination with rehabilitation at the end of the study, and retained them during the follow-up period. Study results are summarized in Table 2.

#### 3.3.1. Functionality of the UL

Among the eight [20,21,22,23,24,26,27,28] studies that analyzed the functionality of the UL, three showed significant improvements [20,21,26], which were maintained for three months [20], six months [21], and one year, respectively [26]. The study by Allman et al. [20] showed significant improvements in the Action research arm test and WMFT by applying 9 sessions of anodic tDCS at a current intensity of 1 mA (35 cm^2^ electrodes, density—0.028 mA/cm^2^) before PT. These results were maintained at the 3-month follow-up, correlating with increased activity during movement of the affected hand and increases in gray matter volume in the ipsilesional motor and promoter cortex in the anodic tDCS stimulation group compared with the control group. The study by Bornheim et al. [21] showed statistically significant improvements after tDCS after applying 20 sessions of anodic tDCS (1 mA/25 cm^2^–0.04 mA/cm^2^) following PT and OT in all motor functional outcomes and somatosensory functions at the 1-year follow-up. The study by Kim et al. [26] showed that at the 6-month follow-up, the cathodic tDCS (20 sessions of anodic tDCS (2 mA/25 cm^2^–0.08 mA/cm^2^) before and after OT) group maintained significant improvements in UEFM compared with the sham group.

#### 3.3.2. Strength of the UL

Among the four studies [22,23,24,25] that analyzed UL strength, the study by Khedr et al. [25] showed significant improvements in force production, as measured by the medical research council scale, which were maintained at the 3-month follow-up after applying 6 sessions of anodic or cathodic tDCS (2 mA/35 cm^2^–0.057 mA/cm^2^) prior to rehabilitation. These findings were related to a greater increase in cortical excitability of the affected hemisphere in the real tDCS versus sham tDCS groups.

#### 3.3.3. Spasticity of the UL

Among the three studies [21,24,27] that analyzed UL spasticity, the study conducted by Bornheim et al. [21] showed significant improvements that were maintained at the 1-year follow-up.

## 4. Discussion

The aim of this review was to establish the long-term maintenance of the effects of tDCS on UL motor performance when applied to stroke patients. When pooled together, the high heterogeneity of stimulation parameters, study designs, and outcome measures make it difficult to draw firm conclusions. So far, tDCS in combination with rehabilitation seems to achieve long-term maintenance of the improvements achieved in UL motor performance in stroke patients, as shown in this review, since for the studies that during its development achieve improvements in the UL motor performance that are maintained at the follow-up time. These results should be taken with caution, since the tDCS technique presents a high interindividual variability of response, as reflected by the similarities of outcome between different real and simulated stimulations. Next, the effect of tDCS and rehabilitation on the maintenance of UL motor improvements in people with stroke will be analyzed according to the characteristics of the subjects and the stimulation parameters.

### 4.1. UL Functionality

The tDCS has so far shown limited effectiveness in poststroke rehabilitation of the UL functionality, being a useful and promising tool in this regard, although with great interindividual variability [4]. The included studies that show improvements in functionality are maintained in the long-term during the follow-up period, raising the question of whether it is necessary to carry out activities to improve functionality after cessation of tDCS, or whether the improvements are maintained without UL rehabilitation.

### 4.2. UL Strength

The application of tDCS has been shown to improve force production in people with stroke, as postulated by Sun et al. [29] in a meta-analysis. In the present review, a study observed that after the improvement in strength production following tDCS and rehabilitation treatment (as measured by the medical research council), these gains were preserved at a 3-month follow-up.

### 4.3. UL Spasticity

One of the studies included in the present review refers to significant improvements in spasticity (assessed by the Modified Ashworth Scale) associated with the application of tDCS and rehabilitation, in addition to the preservation of its long-term effects, but the existing literature states that there is moderate-to-low-quality evidence for no effect of tDCS on improving spasticity in people with stroke [30].

### 4.4. Subject Characteristics

The results do not reflect a clear association between patient profile and maintenance of long-term improvements. The application of tDCS has been shown to be a potentially useful tool in the rehabilitation of all stroke phases (with limited effects so far) [10], with previous studies claiming to find improvements in the functionality of the UL in people with cortical, subcortical, and both-hemisphere (dominant or nondominant) strokes [8]. Therefore, it seems likely that if these patients achieve improvements in UL functionality, such functionality will be maintained over a time span of 3 months to 1 year according to the results of the studies included in the present review. Baseline function may be a limiting factor in achieving UL motor improvements after stroke [31], but not in maintaining these improvements in the long term; four studies were included that showed improvements both at the end of the study and at the end of the follow-up period [20,21,25,26], in three [20,21,26] of which the subjects had high baseline functionality, whereas in one [25] the baseline function was poor. In the four [20,21,25,26] studies the significant improvements achieved were maintained in the long term.

### 4.5. Treatment Characteristics

The type of stimulation analyzed in the present review was anodic and cathodic tDCS, both of which have previously been shown to be beneficial for UL motor rehabilitation in people who have suffered a stroke (especially cathodic stimulation) [32]. These types of stimulation seem to maintain the improvements achieved during tDCS and rehabilitation treatment in the follow-up period. In the case of bi-hemispheric stimulation, it has shown evidence of improvement in UL motor rehabilitation after stroke, where improvements have not yet been shown to be maintained after cessation of tDCS.

The timing of tDCS application is perhaps the least-studied factor with respect to the application protocol. Two models have been established: the Online model, in which tDCS is applied at the same time as rehabilitation, and the Offline model, in which tDCS is applied before rehabilitation. The included studies that maintain long-term improvements apply an Offline model, so they could not be compared in order to establish which is more effective in retaining improvements. In the literature, it seems that the Offline model is more beneficial in terms of improving manual movement accuracy and reaction time applied on the left dorsolateral prefrontal cortex in healthy people [33], so it could be the most recommended model.

The studies included in the present review applied PT and OT, both showing the same results. The type of rehabilitation to be applied seems irrelevant in the long-term maintenance of motor improvements in the UL. What does seem to be decisive is the combination of tDCS and rehabilitation, since the application of tDCS could increase the possible neuroplastic changes derived from rehabilitation. Authors such as Elsner [10] have suggested that an effective motor rehabilitation of the UL in people with stroke is the basis for achieving results after the application of tDCS.

The stimulation parameters of the studies that achieved long-term maintenance of improvements ranged from 20 to 25 min; intensities were 1 or 2 mA, with current densities between 0.028–0.08 mA/cm^2^; the numbers of sessions were 6 [25], 9 [20], 10 [26], and 20 [21]. Stimulation parameters are not comparable due to large heterogeneity. All of these protocols were based on the most widely recommended parameters for UL stimulation according to Jeffery et al. [34]. One factor that could affect the long-term maintenance of motor improvements could be the number of sessions, since these have a nonlinear cumulative effect on the duration of the immediate aftereffects of stimulation. Some animal studies claim that the summation of sessions over time will have a cumulative effect [35,36], with results in human studies suggesting the same [37] and finding evidence that consecutive, daily tDCS sessions have a greater cumulative effect over time than the same number of sessions distributed weekly [38]. However, the studies that maintained improvements in the long term show a disparate number of sessions, ranging from six to 20, so this does not seem to be a determining factor. If motor improvements are achieved after treatment, it seems that these will be maintained regardless of the number of sessions.

### 4.6. Methodological Quality and Risk of Bias

Regarding the evaluation of methodological quality, all the studies included in the review except one [24] had the highest score in the Oxford quality scoring system; thus, they are studies of high methodological quality. The study that obtained a moderate rating was due to performing an inadequate blinding method in the patients, since it did not describe how the sham tDCS was applied. Concerning the assessment of risk of bias, eight studies showed low risk in the Cochrane Library criteria risk of bias tool, while two studies showed performance bias risk. Moreover, two studies showed some concerns due to not mentioning the blinding of the evaluators [22], and not describing how the sham tDCS was applied [24].

### 4.7. Recommendations for Future Research and Clinical Practice

Therefore, one of the implications of our review is that future phase II/III studies of tDCS should collect data at longer follow-up times (greater than 3 months) to gain certainty about the long-term preservation of the significant improvements achieved, as well as the time it takes for such improvements to be lost. Another recommendation for future studies is to clearly indicate whether or not participants receive any type of therapy during the follow-up period. In this way, it will be possible to clarify whether rehabilitation is necessary to maintain the improvements once the application of tDCS has ceased.

### 4.8. Strengths and Limitations

We present a solid systematic review in which we analyze the existing literature on the long-term maintenance of motor improvements in the UL of people with stroke. There has been a large volume of studies evaluating the application of tDCS in stroke, but follow-up time is limited; for this reason, a small number of studies were included in the present review. We were able to extract a general idea of effectiveness without being able to specify which protocol or type of patient is the most appropriate to present these results due to the high heterogeneity between stimulation protocols and the type of patient (stage of stroke, type of stroke, functionality). This review has a good methodological quality, being composed only of RCTs of high methodological quality and low risk of bias.

## 5. Conclusions

It seems that improvements achieved during the application of tDCS and UL motor rehabilitation in stroke patients were preserved at 3-month to 1-year follow-ups.

## Figures and Tables

**Table 1:** Subjects and intervention characteristics.

Study	Population (Number, Men/Women, Age)	Design	Participants’ Characteristics	Protocol	Therapy
Allman et al., 2016 [20]	24 (17/7), 63.5 years	Randomized, double blind, sham-controlled	Chronic, 54.125 months, L/R, cortical and subcortical, 37.66 points	Anodal, 9, daily, 20 min, 1 mA, 35 cm^2^, M1, before rehabilitation	tDCS + PT (40 min daily)
Bornheim et al., 2020 [21]	50 (33/17), 62.98 years	Randomized, triple blind, sham-controlled	Acute, ischemic, Medial Cerebral Artery, Anterior Cerebral Artery, Internal Capsule, L/R, WMFT—47.9 points, handgrip strength—18.06 Kg	Anodal, 20, 5 per week, 20 min, 1 mA, 25 cm^2^, M1, before rehabilitation	tDCS + PT + OT (2 h daily)
Edwards et al., 2019 [22]	82 (50/32), 67.8 years	Randomized, dual-site, double blind, sham-controlled	Chronic, 43.9 months, ischemic stroke, dominant hemisphere, 25.45 points	Anodal, 36, 3 per week, 20 min, 2 mA, 35 cm^2^, M1, before rehabilitation	tDCS + RAT (60 min)
Fusco et al., 2014 [23]	11 (5/6), 58.36 years	Randomized, double blind, sham-controlled	Acute, 19.09 days, L/R, cortical and subcortical (partial anterior circulation, total anterior circulation, lacunar), 24.72 points	Cathodal, 10, daily, 10 min, 1.5 mA, 35 cm^2^, M1, before rehabilitation	tDCS + PT (2 days/week, 45 min)
Hesse et al., 2011 [24]	96 (59/37), 64.97 years	Randomized, double blind, sham-controlled	Subacute, 3.67 months, ischemic stroke, L/R, cortical and subcortical (partial anterior circulation, total anterior circulation, lacunar) 7.97 points	Multimodal, 30, daily, 20 min, 2 mA, 35 cm^2^, M1, during rehabilitation	tDCS + RAT (20 min)
Khedr et al., 2013 [25]	40 (26/14), 58.36 years	Pilot randomized, double blind, sham-controlled	Chronic, 15.6 months, L/R, cortical and subcortical, hand-grip strength—1.67 Kg	Multimodal, 6, daily, 25 min, 2 mA, 35 cm^2^, M1, 1 h before rehabilitation	tDCS + RHB (30 min, daily)
Kim et al., 2010 [26]	18 (13/18), 57.27 years	Prospective, randomized, double blind, sham-controlled	Subacute, 25.43 months, L/R, cortical and subcortical, 37.07 points	Multimodal, 10, daily, 20 min, 2 mA, 25 cm^2^, M1, before and after RHB	tDCS + OT (30 min before and 10 min after)
Koh et al., 2017 [27]	25 (15/10), 56.1 years	Randomized, double blind, sham-controlled	Chronic, 14.6 months, L/R, cortical and subcortical, 23.8 points	Bi-hemispheric, 24, 3 per week, 30 min, 1.5 mA, 25 cm^2^, M1, before RHB	tDCS-SM + OT (20 min) + PT (30 min), 3 times per wek
Triccas et al., 2015 [28]	23 (14/9), 63.4 years	Randomized, double blind, sham-controlled	Subacute and chronic, 31 months, L/R, cortical and subcortical, 19.6 points	Anodal, 18, 2–3 per week, 20 min, 1 mA, 25 cm^2^, M1, before RHB	tDCS + RAT (40 min)

Abbreviations: L/R—Left and right hemisphere; M1—primary motor cortex; mA—Milliamperes; OT—Occupational therapy; PT—Physiotherapy; RAT—Robotic Arm Training; RHB—Rehabilitation; tDCS—transcranial direct current stimulation; tDCS-SM—transcranial direct current stimulation with sensory modulation; WMFT—Wolf Motor Function Test; * No numerical indication of baseline UEFM in the original study.

**Table 2:** Main results of the studies.

Study	Follow-Up	Outcome Measures	Results *			Risk of Bias
Allman et al., 2016 [20]	Three months	Follow-up	Baseline	End of treatment	Last follow-up	Low Risk
		UEFM	Anodal Mean (SD): 38.90 (15.89)	Anodal Mean (SD): 50.36 (11.16)	Anodal Mean (SD): 48.18 (14.35)	
			Sham Mean (SD): 36.42 (17.38)	Sham Mean (SD): 45.54 (14.62)	Sham Mean (SD): 43.15 (16.29)	
		ARAT	Anodal Mean (SD): 20.27 (17.37)	Anodal Mean (SD): 29.91 (21.54)	Anodal Mean (SD): 30.45 (20.92)	
			Sham Mean (SD): 26.27 (20.17)	Sham Mean (SD): 32.54 (21.54)	Sham Mean (SD): 31.31 (21.84)	
		WMFT	Anodal Mean (SD): 38.91 (20.21)	Anodal Mean (SD): 47.18 (17.46)	Anodal Mean (SD): 48.36 (18.19)	
			Sham Mean (SD): 39.65 (25.39)	Sham Mean (SD): 48.00 (23.42)	Sham Mean (SD): 43.09 (23.78)	
Bornheim et al., 2020 [21]	One year	UEFM	Main effect for time: F = 173.1, *p* = 0.0001 Main effect for treatment: F = 2.5, *p* = 0.123 Time-by-treatment interaction: F = 28, *p* = 0.0001			Low Risk
		WMFT	Main effect for time: F = 358.8, *p* = 0.0001 Main effect for treatment: F = 6.6, *p* = 0.015 Time-by-treatment interaction: F = 56.6, *p* = 0.0001			
Edwards et al., 2019 [22]	Six months	UEFM	Anodal Mean (SD): 25.7 (16.3)	Anodal Mean (SD): 32.0 (18.8)	Anodal Mean (SD): 32.3 (18.8)	Some concerns
			Sham Mean (SD): 25.3 (16.3)	Sham Mean (SD): 33.4 (19.2)	Sham Mean (SD): 35.1 (19.3)	
		WMFT	Anodal Mean (SD): 56.0 (47.2)	Anodal Mean (SD): 68.5 (23.2)	Anodal Mean (SD): 72.7 (54.5)	
			Sham Mean (SD): 60.0 (48.3)	Sham Mean (SD): 67.1 (54.0)	Sham Mean (SD): 51.8 (57.8)	
		MRC	No mean difference reported			
Fusco et al., 2014 [23]	Three months	UEFM	T1–T0 changes Cathodal Mean (SD): 4 (5); *p* = 0.045 Sham Mean (SD): 4 (7); *p* = 0.003			Low Risk
		MF	Main effect for time: *p* = 0.130 Main effect for treatment: *p* = 0.612 Time-by-treatment interaction: *p* = 0.882			
		9HPT	Main effect for time: *p* = 0.007 Main effect for treatment: *p* = 0.655 Time-by-treatment interaction: *p* = 0.372			
Hesse et al., 2011 [24]	Three months	UEFM	Anodal Mean (SD): 7.8 (3.8)	Anodal Mean (SD): 19.1 (14.4)	Anodal Mean (SD): 23.2 (18.3)	Some concerns
			Cathodal Mean (SD): 7.9 (3.4)	Cathodal Mean (SD):18.9 (10.5)	Cathodal Mean (SD): 23.5 (14.5)	
			Sham Mean (SD): 8.2 (4.4)	Sham Mean (SD): 19.2 (15.0)	Sham Mean (SD): 22.5 (17.1)	
		MRC	Anodal Mean (SD): 3.5 (3.6)	Anodal Mean (SD): 11.9 (12.5)	Anodal Mean (SD): 11.7 (14.4)	
			Cathodal Mean (SD): 2.9 (3.4)	Cathodal Mean (SD): 13.7 (10.4)	Cathodal Mean (SD): 13.5 (10.3)	
			Sham Mean (SD): 3.4 (3.2)	Sham Mean (SD): 12.8 (12.1)	Sham Mean (SD): 13.5 (14.3)	
		MAS	Anodal Mean (SD): 1.6 (2.9)	Anodal Mean (SD): 3.3 (3.6)	Anodal Mean (SD): 3.6 (6.9)	
			Cathodal Mean (SD): 1.0 (1.8)	Cathodal Mean (SD): 3.5 (4.9)	Cathodal Mean (SD): 3.5 (5.0)	
			Sham Mean (SD): 1.4 (2.7)	Sham Mean (SD): 3.5 (4.0)	Sham Mean (SD): 3.8 (5.5)	
Khedr et al., 2013 [25]	Three months	Hand-grip strength	No mean difference reported, *p* = 0.175			Low Risk
Kim et al., 2010 [26]	Six months	UEFM	Main effect for time: F = 16.95, *p* < 0.001 Main effect for treatment: F = 0.65, *p* = 0.537 Time-by-treatment interaction: F = 3.55, *p* = 0.017 Cathodal: *p* < 0.05			Low Risk
Koh et al., 2017 [27]	Three and six months	UEFM	Bi-hemispheric Mean (SD): 20.4 (6.2)	Bi-hemispheric Mean (SD): 6.0 (1.5)	Bi-hemispheric Mean (SD): 4.3 (1.5)	Low Risk
			Sham Mean (SD): 27.2 (9.4)	Sham Mean (SD): 1.3 (1.8)	Sham Mean (SD): 0.2 (1.7)	
		ARAT	Bi-hemispheric Mean (SD): 2.1 (2.1)	Bi-hemispheric: 0.5 (0.5).	Bi-hemispheric: 0.7 (0.6)	
			Sham: 4.7 (9.1)	Sham: 0.0 (0.6)	Sham: −0.7 (0.7).	
		MAS (Elbow flexion)	Bi-hemispheric Mean (SD): 1.4 (0.7)	Bi-hemispheric Mean (SD): −0.1 (0.1)	Bi-hemispheric Mean (SD): 0.1 (0.1)	
			Sham Mean (SD): 1.3 (0.3)	Sham Mean (SD): −0.2 (0.2)	Sham Mean (SD): −0.1 (0.2)	
Triccas et al., 2015 [28]	Three months	UEFM	Anodal Mean (SD): 24.91 (16.01)	Anodal Mean (SD): 33.64 (16.25)	Anodal Mean (SD): 32.09 (16.65)	Low Risk
			Sham Mean (SD): 37.09 (13.57)	Sham Mean (SD): 44.82 (16.29)	Sham Mean (SD): 44.18 (18.08)	
		ARAT	Effect Of time: X^2^ = 16.636, df = 2, *p* < 0.001 Effect of group: X^2^ = 1.403, df = 1, *p* = 0.236 Time-by-group: X^2^ = 2.293, df = 1, *p* = 0.130			

* Data is presented as mean (standard deviation) unless otherwise specified. Abbreviations: 9HPT—9-hole peg test; ARAT—Action Research Arm Test; MAS—Modified Ashworth Scale; MF—manual force; MRC—Medical Research Council; UEFM—Fugl-Meyer upper extremity score; WMFT—Wolf motor function test.

## Data Availability

All the papers included in this systematic review have been downloaded from the original publisher sites and are available to any researcher. Some of the publisher platforms may require a subscription to be able to read and download the papers.

## Supplementary Materials

The following are available online at https://www.mdpi.com/article/10.3390/s21155216/s1. Table S1: PRISMA checklist. Table S2: Complete bibliographic search. Table S3: Oxford quality scoring system.

## Abbreviations

NIBS	Noninvasive brain stimulation techniques
RCT	Randomized controlled trial
OT	Occupational therapy
PT	Physiotherapy
tDCS	Transcranial direct current stimulation
UEFM	Fugl-Meyer Upper Extremity Test
UL	Upper limb
WMFT	Wolf Motor Function Test
